# Prevalence, Risk Factors, and Diagnostic Efficacy of Bovine Tuberculosis in Slaughtered Animals at the Chiang Mai Municipal Abattoir, Thailand

**DOI:** 10.3389/fvets.2022.846423

**Published:** 2022-03-29

**Authors:** Tawatchai Singhla, Sukolrat Boonyayatra

**Affiliations:** ^1^Department of Food Animal Clinic, Faculty of Veterinary Medicine, Chiang Mai University, Chiang Mai, Thailand; ^2^Center of Excellence in Veterinary Public Health, Faculty of Veterinary Medicine, Chiang Mai University, Chiang Mai, Thailand

**Keywords:** bovine tuberculosis, prevalence, risk factors, diagnostic efficacy, abattoir, Thailand

## Abstract

This study aimed to (1) investigate the prevalence of bovine tuberculosis (bTB) in slaughtered animals at the Chiang Mai Municipal abattoir in Chiang Mai, Thailand; (2) identify animal-level risk factors for bTB at the abattoir; and (3) evaluate the performance of techniques for bTB detection at the abattoir. From April 2020 to March 2021, 161 animals registered for slaughter were randomly selected for the study. Animal data including age, sex, species, body condition scores, and origins of the animals were collected. Meat inspection was performed by a trained meat inspector. Tissue samples of the lung, liver, and lymph nodes were collected for histopathological diagnosis and polymerase chain reaction (PCR) detection of Mycobacteria and specifically *Mycobacterium bovis*. The prevalence of bTB during meat inspection and PCR was calculated separately. Animal-level factors affecting bTB were determined using multivariate logistic regression analysis. The performance of meat inspection and PCR was evaluated using a Bayesian approach. The prevalence of bTB was 12.4% (20/161) and 34.8% (56/161) when the disease was diagnosed using meat inspection and PCR, respectively. Buffaloes had a significantly higher risk of being identified as bTB-positive using PCR compared to beef cattle (odds ratio = 2.19; confidence interval = 1.11–4.30). The median of posterior estimates of sensitivity (Se) and specificity (Sp) to detect bTB using meat inspection were 20.8% [95% posterior probability interval (PPI) = 9.1–36.5%] and 87.8% (95% PPI = 79.6–95.4%), respectively. The medians of the posterior estimates of Se and Sp for PCR were 88.6% (95% PPI = 70.5–98.3%) and 94.4% (95% PPI = 84.7–98.8%), respectively. These findings demonstrate that bTB is highly prevalent among slaughtered animals. PCR can be used as an ancillary test for bTB surveillance at abattoirs in Thailand.

## Introduction

Bovine tuberculosis (bTB), a chronic disease in cattle and buffaloes, is caused by members of the *Mycobacterium tuberculosis* complex (MTBC), including *Mycobacterium tuberculosis, Mycobacterium bovis, Mycobacterium orygis, Mycobacterium caprae, Mycobacterium microti, Mycobacterium pinnipedii, Mycobacterium mungi*, and *Mycobacterium suricattae* (1). MTBC can cause tuberculosis in mammals, including domestic and wild animals, and humans *M. bovis* is the most common species reported to cause bTB in domestic cattle and buffaloes. In addition to *M. bovis*, other members of MTBC such as *M. tuberculosis* (2, 3), *M. caprae* (4), *M. orygis* (5), *M. pinnipedii* (6), and *M. microti* (7), have also been reported to cause bTB in cattle. Cattle and buffaloes are susceptible to *M. bovis*. The pathogen can spread to humans primarily through the consumption of milk and meat from infected animals as a zoonotic disease and kills individuals annually (8). Therefore, the disease is a critical public health burden and causes severe economic losses due to impairment of animal health, production losses, costs of eradication programs, and trade restrictions (9). Annual agricultural losses due to bTB have been estimated to be ~3 billion United States Dollars worldwide (10).

Risk factors associated with bTB outbreaks have been reported at different levels (11). Several studies have previously identified the animal-level risk factors for bTB outbreaks. These factors include age, sex, breed, body weight, average daily gain, immune status, genetic resistance or susceptibility to bTB, vertical and pseudo-vertical transmission, and auto contamination (12–15). The age of animals is a major individual factor that has been identified as a risk in both developed and developing countries (16, 17). A study in Ireland in 1996 found that adult cattle were more likely to be infected with *M. bovis* than younger calves (16). Therefore, it has been hypothesized that exposure duration is positively associated with age.

The bTB eradication and control program is based on the test and slaughter policy and/or surveillance at abattoirs. The abattoir surveillance system is primarily performed by detecting suspected granulomatous lesions during the slaughtering process. The bTB positive animal is traced back to the farm of origin for further bTB investigations through a tuberculin skin test (18–20). The abattoir surveillance system is used as an ancillary method for live animal testing to increase the chance of disease detection in both officially bTB-free (OTF) and non-OTF countries (18). However, the sensitivity (Se) and specificity (Sp) of detecting lesions during meat inspection at abattoir surveillance vary, ranging from 25 to 96% and 22 to 100%, respectively (20–23).

To enhance the performance of a bTB surveillance program at abattoirs, additional assays, such as PCR, can be used to confirm suspected lesions from a meat inspection process (19). PCR has also been implemented for investigations of bTB in slaughtered animals at abattoirs in many countries (24, 25). The performance of PCR has been reported with an Se and Sp of 87.7% (94.3–99.0%) and 93.6% (89.9–96.9%), respectively (19).

Thailand launched a bTB eradication program using a test and slaughter policy for a decade. However, this program has only been implemented in dairy cattle. Beef cattle and buffaloes were not required to participate in the control program. Therefore, this disease has not been eradicated. The bTB is considered a neglected zoonotic disease in Thailand due to its low prevalence in live adult dairy cattle. The bTB surveillance system at abattoirs, which primarily slaughter beef cattle and buffaloes, is rarely performed. Thus, information on bTB at abattoirs, such as prevalence, risk factors, and the efficacy of bTB detection is also scarce. This study aimed to (1) investigate the bTB prevalence in beef cattle and buffaloes at the Chiang Mai Municipal abattoir; (2) identify animal-level risk factors for bTB; and (3) evaluate the performance of meat inspection and PCR for the detection of bTB at abattoirs using a Bayesian approach.

## Materials and Methods

### Study Area and Sample Collection

This cross-sectional study was performed at the Chiang Mai Municipal abattoir, Chiang Mai, Thailand. This Chiang Mai Municipal abattoir is the only abattoir certified for compliance with the Thai agricultural standard on good manufacturing practices (TAS-GMP) for cattle and buffalo abattoir (TAS 9019-2007) in Chiang Mai province. At the abattoir, there was only one single person who was officially trained to be a meat inspector, which is a minimal requirement according to the TAS-GMP standard (26). A sample size required for a population survey was estimated using Epi Info™ version 7.2.3.1 (27) with the following parameters: (1) a population of 800 slaughtered animals in the Chiang Mai Municipal abattoir, (2) expected bTB prevalence of 14% as previously estimated by Singhla et al. (28), and (3) the accepted margin of error of 5%. From the calculation, the minimal required sample size for the current study was 150 animals. Between April 2020 and March 2021, 15 animals registered for slaughtering each week were inspected by a single trained meat inspector, and 3–4 animals per week were randomly selected. Information on slaughtered animals was collected from the abattoir database, including the type of animals, sex, age, body condition score, the origin of animals, and date of slaughter. During the meat inspection process, suspected organs and carcasses with bTB-like lesions were recorded. Tissue samples from the lungs; liver; and lymph nodes such as retropharyngeal, mediastinal, trachea-bronchial, and mesenteric, were aseptically collected from the selected carcasses and sent to the Veterinary Diagnostic Laboratory at the Faculty of Veterinary Medicine, Chiang Mai University for *M. bovis* identification using PCR. The animal study was reviewed and approved by the Animal Use Ethics Committee of the Faculty of Veterinary Medicine, Chiang Mai University (R10/2563).

### Meat Inspection

The meat inspection procedure was performed on the intact visceral organs of each of the selected carcasses, particularly the lungs, liver, gastrointestinal tract, and lymph nodes. Each organ was assessed macroscopically by visual inspection, palpation, and incision. All tissue samples of the lymph nodes, lungs, and liver from the carcasses were collected by a single meat inspector of the Chiang Mai Municipal abattoir. During the inspection process, the organs were examined to detect the presence of suspect bTB lesions. The carcasses with tubercle formation, such as an abscess with necrotic focus and caseation and sometimes calcification surrounded by a fibrous capsule, were defined as suspected bTB on meat inspection (29).

### DNA Extraction

Tissue samples from each animal were pooled for DNA extraction. Pooled tissue samples were homogenized. A mixture of 300 mg of homogenate with 250 μL of distilled water and 250 μL of lysis buffer was added to a 2-mL tube containing 300 mg of 0.5-mm glass beads and subjected to mechanical disruption at 30 Hz for 20 min. After mechanical lysis of the tissue, DNA was extracted using NucleoSpin® Tissue (MACHEREY-NAGEL, Duren, Germany) according to the manufacturer's instructions.

### Genus Identification of *Mycobacterium* spp.

The 16S rRNA gene (1,030 base pairs) of *Mycobacterium* spp. was amplified using PCR from the extracted DNA. Each PCR reaction contained 32.8 μL of distilled water, 0.2 μL of Taq polymerase buffer (5 U/μL), 3 μL of MgCl_2_ (25 mM), 1 μL of dNTPs (10 mM), 2 μL of 16S rRNA MYCGEN-F primer (5′-AGAGTTTGATCCTGGCTCAG-3′) (10 μM), 2 μL of 16S rRNA MYCGEN-R primer (5′-TGCACACAGGCCACAAGGGA-3′) (10 μM), 5 μL of 10X PCR Buffer, and 5 μL of template DNA. A thermocycler was used for amplification with the following cycling program: 5 min of initial denaturation at 95°C, followed by 35 cycles of 30 s of denaturation at 95°C, 30 s of annealing at 55°C, and 45 s of extension at 72°C, and a final extension at 72°C for 7 min. The PCR products were separated by electrophoresis on a 2% agarose gel and visualized using an ultraviolet illustrator. Positive samples were considered to contain bacteria in the genus *Mycobacterium*.

### Detection of MTBC and *M. bovis*

To identify *M. bovis*, the PCR products of the 16S rRNA gene PCR-positive samples were subsequently tested using multiplex PCR for identification of *M. bovis*, as described previously (30). Multiplex PCR was performed with specific primers for the MTBC using TB1 (5′-CCTGCGAGCGTAGGCGTCGG-3′) and TB2 (5′-CTCGTCCAGCGCCGCTTCGG-3′), and *M. bovis-specific* primers using pncATB-1.2 (5′-ATGCGGGCGT TGATCATCGTC-3′) and pncAMB-2 (5′-CGGTGTGC CGGAGAAGCCG-3′). The multiplex PCR was performed by mixing 1 μL of DNA extracts from all mycobacterial reference strains and tissue samples with 24 μL of reaction mixture containing 0.2 pmol μL TB1 and TB2 primers, 0.4 pmol μL pncATB-1.2 and pncAMB-2 primers and other PCR mixtures as described in the previous section. Similar PCR cycles were conducted for amplification as described in the previous section (31–33).

### Statistical Analysis

The apparent prevalence of bTB in meat inspections and PCR was calculated separately. The animal-level risk factors were identified using logistic regression analysis. The analyses were divided into two stages and implemented in R version 4.1.0 (34). In the first stage, categorical and continuous variables were primarily screened using univariate logistic regressions via the lme4 package (35). Multicollinearity among predictor variables was evaluated using the chi-square test for categorical variables and Pearson product-moment correlation of continuous variables (cor ≥ 0.5). If multicollinearity was identified (*P* < 0.05, or cor ≥ 0.5), the variable with higher biological plausibility was offered to the next stage.

In the second stage, variables from the previous analysis (*P* ≤ 0.2 and without marked multicollinearity among the candidate variables were included in the full multivariate logistic regression for model selection. A stepwise procedure based on the Akaike information criterion (AIC) was performed using the bestglm package (36). The final model was selected from the model with the lowest AIC value. If several candidate models had similarly low AIC values (difference in AIC value <2), the model with the fewest parameters was preferred as the final model because of the principle of parsimony.

Cohen's kappa analysis was conducted to assess the agreement between the meat inspection and PCR results. The analysis results were categorized into six categories based on kappa values (0–1) into slight (0–0.20), fair (0.21–0.40), moderate (0.41–0.60), substantial (0.61–0.80), and almost perfect (0.80–1.0) agreements (37). Regarding the imperfect characteristics of both meat inspection and PCR from tissue samples, the performance of meat inspection and PCR for the detection of bTB in carcasses and the true prevalence of the disease were analyzed using a Bayesian latent class modeling approach as described by Joseph et al. (38). The Bayesian model assumes that for the *k* populations, the counts (Y_*k*_) of the different combinations of test results, such as +/+, +/–, –/+, and –/– for two tests follow a multinomial distribution: Y_*k*_ | P_*qrk*_ ~ multinomial (n_*k*_, {P_*qrk*_}), where *qr* is the multinomial cell probability for the two-test outcome combination, and P*qrk* is a vector of probabilities of observing the individual combinations of test results. The priors for Se and Sp of both meat inspection and PCR and priors for bTB prevalence rates were derived from previous studies and modeled as beta distributions (19–23, 28). All the priors are listed in Table 1. Meat inspection is based on indirect detection of bTB, such as granulomatous lesions in visceral organs, whereas PCR is based on direct detection of the pathogen in clinical samples. Therefore, a Bayesian model for two conditionally independent tests was implemented in a single population to evaluate the Se and Sp of each test and estimate the true disease prevalence. All analyses were performed in JAGS 4.3.0 via the rjags and R2jags packages from R version 4.1.0 (34, 39, 40). Posterior distributions were computed after 100,000 iterations of the models with the first 10,000 discarded as the burn-in phase.

**Table 1:** Prior estimates for mode and 95% confidence interval (CI) for sensitivity and specificity values of meat inspection and polymerase chain reaction (PCR) test and prevalence of the disease (%).

**Diagnostic tests**	**Parameters**	**Mode**	**95% CI^a^**
Meat inspection^b^	Sensitivity	84.0	>35.0
	Specificity	41.0	>12.0
PCR^c^	Sensitivity	87.0	>52.0
	Specificity	97.0	>90.0
Disease prevalence		10.0	<20.0

a *95% lower or upper credibility interval bound*.

b *Meat inspection*.

c *PCR*.

The model convergence was tested by the Gelman-Rubin diagnostic plot visual inspection using three sample chains with different initial values (41). Model sensitivity was analyzed to evaluate the influence of prior information and the assumption of conditional dependence between meat inspection and PCR on the posterior estimates (42). For these analyses, each prior was replaced by a non-informative uniform 0–1 distribution, and the changes in the model were subsequently considered as appreciable effects of priors on the model (change > 25% of median value). Then, the deviance information criteria (DICs) of the models with and without the covariance term were compared. The model with the lowest DIC was preferred as the final model (41).

## Results

### Prevalence of bTB at Abattoir

Throughout the study period, 161 animals, including 72 beef cattle and 89 buffaloes, were randomly selected from a total of 780 slaughtered animals. The average age of the sampled animals was 4.7 years. Most of them were females with a body condition score >3 (83.2%). Most beef cattle and buffaloes were from live animal markets located in Chiang Mai province, with percentages of 58.3 and 78.7%, respectively. No clinical signs of any disease were observed in any of the animals before slaughter.

The prevalence of bTB among slaughtered animals was 12.4% (20/161) and 34.8% (56/161) when the disease was diagnosed by meat inspection and PCR, respectively (Table 2). From meat inspection, three of 72 beef cattle (4.2%) and 17 of 89 buffaloes (19.1%) were considered positive. Regarding PCR results, 56 carcasses, including 17 of 72 beef cattle (23.6%) and 39 of 89 buffaloes (43.8%), suspected to be positive for the *Mycobacterium* genus were subsequently identified as having the *Mycobacterium tuberculosis* complex. However, PCR products specific for *M. bovis* was not detected from any tissue sample.

**Table 2:** Cross-classified test results for bovine tuberculosis in 161 animals from meat inspection and polymerase chain reaction (PCR) tests.

**Diagnostic test results**		**PCR**		**Total**
		**Positive**	**Negative**	
Meat inspection	Positive	8	12	20
	Negative	48	93	141
	Total	56	105	161

### Factors Affecting bTB Status of Animals Detecting by PCR

For univariate analysis, two variables, including animal type and animal age, were significantly associated with bTB status (*P* ≤ 0.2). No multicollinearity was observed among the variables (Table 3). Both variables were included in the multivariate logistic regression analysis of the final model.

**Table 3:** Univariate analysis of variables as *P* ≤ 0.2 considered for multivariate analysis.

**Variables**	**Mean** **±SE or percentage**		**Coefficient**	***P*-value**
	**Positive**	**Negative**		
Types of animals			0.78	0.023
- Buffalo	43.8%	56.2%		
- Beef cattle	23.6%	76.4%		
Age of animals	4.9 ± 0.9%	4.5 ± 0.9%	0.33	0.067

Regarding the multivariate logistic regression analysis, the type of animal (beef cattle vs. buffaloes) was the only variable remaining in the final model. The buffaloes had a significantly higher risk of bTB positive for PCR than beef cattle (*P* < 0.05), as shown in Table 4.

**Table 4:** Final multiple logistic regression models to find factors affecting bovine tuberculosis (bTB) in animals at the Chiang Mai Municipal abattoir, Thailand.

**Variables**	**β^a^**	**SE^b^**	**OR^c^**	**CI^d^**	***P*-value**
Types of animals (buffalo)	0.78	0.35	2.2	1.1–4.3	0.023

a *Regression coefficient*.

b *Standard error*.

c *Odds ratio*.

d *95% confidence interval*.

### Performance of Meat Inspection and PCR for the Diagnosis of bTB

The agreement between meat inspection and PCR was slight (Kappa = 0.03). When Bayesian latent class modeling was applied to determine Se and Sp of both techniques, the median of Se estimates for meat inspection was 20.8 with a 95% posterior probability interval (PPI) ranging between 9.1 and 36.5% and much lower than its prior values. Conversely, the estimated Sp of the meat inspection was 87.8% (95% PPI = 79.6–95.4%) and higher than its prior estimate.

The posterior estimates of Se and Sp for PCR were 88.6% (PPI = 70.5–98.3%) and 94.4% (PPI = 84.7–98.8%), respectively, and they were close to their prior values. The posterior estimate for the disease prevalence was higher than the prior estimate with a median value of 30.1% (PPI = 22.1–40.4%), as shown in Table 5.

**Table 5:** Posterior estimates for median and 95% posterior probability interval (PPI) for sensitivity and specificity of meat inspection and polymerase chain reaction (PCR) test for the diagnosis of bovine tuberculosis, and prevalence of the disease.

**Diagnostic tests**	**Parameters**	**Median (%)**	**95% PPI^a^ (%)**
Meat inspection	Sensitivity	20.8	9.1–36.5
	Specificity	87.8	79.6–95.4
PCR	Sensitivity	88.6	70.5–98.3
	Specificity	94.4	84.7–98.8
Disease prevalence		30.1	22.1–40.4

a *95% posterior probability interval*.

According to the model selection, the conditional dependent model, with a covariance term between meat inspection and PCR, showed a lower DIC value compared to the conditional independent model (22.8 vs. 30.6%, respectively). Thus, the conditional dependent model was selected as the final model. However, the conditional covariance of the model between the meat inspection and PCR was low in both infected and uninfected animals, and the probability intervals of the conditional covariance included 0, which indicated that the correlation between the results of both tests was low.

After the burn-in phase, the model converges properly without autocorrelation. There was no appreciable effect on sensitivity analyses (change > 25% of median value) in the posterior estimates of the Se and Sp of PCR, and the Sp of the meat inspection technique when non-informative distributions were used as priors for any parameter. This result is interpreted as evidence of model robustness. However, a larger change in posterior estimates for the Se of the meat inspection (from 20.8 to 13.5%) was observed when a non-informative prior was used. This finding suggests that the prior of this parameter influences the modeling process.

## Discussion

This study reported the prevalence of bTB at the abattoir, as determined by meat inspection and PCR techniques. The prevalence of the disease using PCR was higher than that of meat inspection. The results agree with a study in Ecuador, which reported a higher prevalence of bTB when PCR was used for disease detection compared with meat inspection (17). This finding indicated low sensitivity of the meat inspection because most infected animals are in the early stage of infection or lesions are very small or invisible (43). However, the presence of bTB in animals at the abattoir should be documented, raising concerns regarding zoonotic diseases or food safety in this region.

In the current study, MTBC were reported to be detected from beef cattle and buffaloes slaughtered at the Chiang Mai Municipal abattoir in Thailand. In contrast, a previous study reported all negative results of bTB among swamp buffaloes in northeastern Thailand using the comparative intradermal test, which is the test to detect the MTBC infection in lived animals (44). Our finding suggests that other members of MTBC rather than *M. bovis* might be the cause of bTB among beef cattle and buffaloes in northern Thailand. Several studies demonstrated the infections of different members of MTBC in cattle worldwide, such as *M. caprae* in Spain (4), *M. microti* in France (7), *M. orygis* in Bangladesh (5), and *M. tuberculosis* in several countries such as in China (2), India (45), and South Africa (3). Infections of *M. tuberculosis* in domestic animals have been increasingly concerned as a reverse zoonosis. A study in India reported a higher prevalence of *M. tuberculosis* in cattle than *M. bovis*, which was probably due to the reverse transmission from human to cattle in the endemic areas of human TB (46). In Thailand, a previous study reported *M. tuberculosis* infections in domesticated elephants, suggesting the possibility of a reverse zoonotic transmission from human to animal in the country (47). Findings of the present study did not provide the species identification of MTBC to confirm the existing of reverse zoonoses in beef cattle and buffaloes in Thailand, which should be further investigated in the future study.

The higher prevalence of bTB in buffaloes compared to beef cattle is consistent with previous reports (48, 49). Accordingly, buffaloes posed a higher risk of bTB than beef cattle. This might be explained by the different behaviors of buffaloes and beef cattle. Buffaloes are very social and are likely to aggregate in pastures (48). Moreover, compared to cattle, buffaloes are better adapted to protect themselves from heat than cattle by spending a lot of time wallowing in the mud to reduce heat stress. This could be a potential factor for the spread of the pathogen within the herd (49). Another reason might be the differences in herd management practices between buffalo and beef cattle. In Thailand, buffaloes are typically raised inattentively. For example, buffaloes are generally fed with poor-quality feed and raised without permanent stability. Moreover, genetic differences associated with susceptibility to bTB between buffaloes and beef cattle have never been studied and should be clarified.

According to the TAS-GMP for cattle and buffalo abattoir (TAS 9019-2007), meat inspection by visual inspection, palpation, and incision of visceral organs, is performed in order to ensure the food safety for the consumers. It is not specifically designed for the bTB diagnosis. Biffa et al. demonstrated that the routine abattoir meat inspection had lower efficiency to detect bTB compared to detailed abattoir meat inspection procedures (43). Therefore, only routine abattoir meat inspection is not sufficient for the effective bTB surveillance program. Our findings revealed that the bTB detection results using meat inspection were in poor agreement with those obtained using PCR. A previous study in 2013 also demonstrated a low agreement of bTB diagnosis between meat inspection and bacterial culture (22). Moreover, the conditional dependence of the final model between meat inspection and PCR was low in both infected and non-infected animals, indicating that the test results were independent of each other. The lack of correlation between the test outcomes suggests that their application as parallel tests would help to increase the performance of the surveillance strategy in current bTB eradication programs (50).

The current study reported low Se in meat inspection at the abattoir. This finding agrees with the findings of previous studies (22, 23). A previous study at the municipal abattoir in Ethiopia reported that the Se of meat inspection was only 25% when the bacterial culture was used as a reference test (22). The low Se of meat inspection might be due to the inability to identify visible lesions from early infected animals, the limited time available for each tissue inspection in the processing line, the limitations of inspection facilities (e.g., light intensity, working space and time), and the low experience of meat inspectors (20, 51, 52). Moreover, infections with other members of the MTBC rather than *M. bovis* may have a lower propensity to cause classical tuberculous lesions in infected cattle. In contrast, the posterior estimates of Sp of meat inspection reported in the present study were higher than those reported in several previous studies, which reported a median Sp ranging between 72 and 77% (20, 22). However, a meta-analysis study in the United Kingdom (UK) and Ireland reported a higher Sp of meat inspection, ranging from 99 to 100% (23). These variations in estimating the Sp of meat inspection may be related to the varied experience of meat inspectors and the difference in the reference tests used to compare with the meat inspection (20). For instance, a study in Ethiopia reported two different Sp estimates of meat inspection to be 72 and 75% when bacterial isolation and PCR were used as the reference tests, respectively (20).

Posterior estimates for Se and Sp of PCR were estimated to be higher than those of meat inspection, which is consistent with previous studies (12, 16). PCR can detect live or dead mycobacteria at all stages of infection. Therefore, the Se of PCR is not influenced by the presence or absence of lesions on animal carcasses (19, 53, 54). However, a study in Spain reported a low Se of PCR (61.1%) when it was implemented in samples without tuberculosis-like lesions (55). Therefore, PCR can be used together with meat inspection to improve the efficiency of bTB detection at abattoirs.

In this study, the posterior estimate of the true prevalence of bTB was quite high. This might be because the test and cull policies have never been implemented for buffaloes and beef cattle in Thailand. Therefore, infected animals are not removed from herds and consequently sent to the abattoirs, leading to an increased chance of bTB detection. Moreover, it has been reported that there are substantial informal trades in live cattle and buffaloes between Thailand and neighboring countries (56). This can increase the risk of bTB outbreaks and the disease prevalence in the country. However, the inferences based on the findings from this study should be cautiously made. The Chiang Mai Municipal abattoir is the only abattoir with a trained meat inspector in Chiang Mai province. To survey the bTB using meat inspection, only animals slaughtered at this particular abattoir were included in the study. Therefore, the estimated prevalence of the selected population might be different from true prevalence estimated from the population of all slaughtered beef cattle and buffaloes in this region due to the selection bias.

## Conclusion

This study provides the prevalence and risk factors of bTB in animals at the Chiang Mai Municipal abattoir, Thailand. The estimated prevalence of bTB among slaughtered animals was quite high. Buffaloes had a higher risk of bTB infection than beef cattle. The Se and Sp of the PCR were higher than those of meat inspection for the detection of bTB. This finding suggests that PCR can be used as an ancillary test with meat inspection to improve the efficiency of bTB detection in slaughtered animals in an abattoir surveillance system.

## Data Availability Statement

The raw data supporting the conclusions of this article will be made available by the authors, without undue reservation.

## Ethics Statement

The animal study was reviewed and approved by the Animal Use Ethics Committee of the Faculty of Veterinary Medicine, Chiang Mai University (R10/2563).

## Author Contributions

TS and SB designed the study and wrote the manuscript. TS conducted the laboratory work and analyzed the data. Both authors read and approved the final manuscript.

## Funding

This work was funded by Chiang Mai University (R000018559, 2020).

## Conflict of Interest

The authors declare that the research was conducted in the absence of any commercial or financial relationships that could be construed as a potential conflict of interest.

## Publisher's Note

All claims expressed in this article are solely those of the authors and do not necessarily represent those of their affiliated organizations, or those of the publisher, the editors and the reviewers. Any product that may be evaluated in this article, or claim that may be made by its manufacturer, is not guaranteed or endorsed by the publisher.
